# High blood pressure in the emergency department as an opportunistic screening tool for detection of hypertension

**DOI:** 10.1038/s41371-024-00977-4

**Published:** 2024-11-16

**Authors:** Sara Bentzel, Karin Manhem, Ottilia Öhman, Karzan Abdulla, Georgios Mourtzinis

**Affiliations:** 1 Department of Molecular and Clinical Medicine, Institute of Medicine, Sahlgrenska Academy, University of Gothenburg, Gothenburg, Sweden; 2 Department of Cardiology, Sahlgrenska University Hospital, Gothenburg, Sweden; 3 Department of Medicine and Emergency Mölndal, Sahlgrenska University Hospital, Gothenburg, Sweden

**Keywords:** Hypertension, Diagnosis

## Abstract

Hypertension is the most preventable cause of morbidity and mortality, but many individuals are underdiagnosed and lack treatment control. High blood pressure (BP) in the emergency department (ED) is commonly observed, but mostly used for short-term evaluation. We aimed to study the usefulness of high BP in the ED as a screening tool for undiagnosed hypertension. We used the electronic medical record system to identify all patients that had attended the ED at a university hospital from 2018-01-01 to 2018-03-31 and from 2018-07-01 to 2018-09-30 with an obtained systolic BP ≥ 160 and/or diastolic BP ≥ 100 mmHg measured at the ED. We excluded patients with previously diagnosed hypertension and patients on BP-lowering medication. All patients identified where contacted two years after attending the ED, with a letter of consent and a questionnaire regarding diagnosis of hypertension and current medication. 5424 patients attended the ED during the 6-months-period. 271 patients met the inclusion criteria and were asked to participate. 167 individuals (62%) agreed to participate and responded to the questionnaire. Mean age of participants were 63.1 years and 51% were women. 134 patients (80%) had measured their BP after the ED-visit, and 48 (36%) of those had been diagnosed with hypertension. 96% of patients diagnosed with hypertension were on BP-lowering medication. To follow-up BP ≥ 160/100 mmHg after an ED visit can reveal undiagnosed hypertension in one third of the patients. Given the amount of undiagnosed hypertension, an ED-measured BP might provide an important tool to detect and start treatment of hypertension.

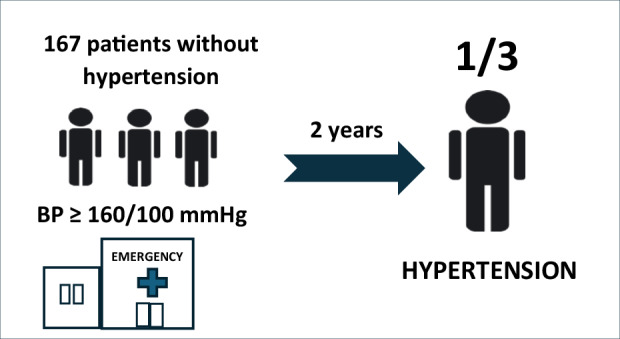

## Introduction

Hypertension is the most preventable cause of morbidity and mortality, and early detection and treatment initiation is crucial. Despite this, hypertensive patients are often underdiagnosed and many lack treatment control [1–3] why opportunistic screening could be valuable [4]. However, screening for hypertension is debated and a recent Cochrane report stated that evidence to support any screening strategy is lacking and called for further randomized or observational studies to diminish the knowledge gap [5].

In the emergency department (ED), blood pressure (BP) is measured on almost every patient to assess the patient’s condition and acuity, regardless of the presenting complaint [6, 7]. Previous reports have shown that high BP is commonly observed in the ED, but the results are mostly used for short-term evaluation [8, 9]. Since BP-measurements in the ED may be affected by stressors such as pain and anxiety, there is a considerable debate whether these measurements accurately reflect an individuals’ true BP and raises the question of its predictive values. A recent study, however, showed a strong association between a first BP obtained in the ED and arteriosclerotic cardiovascular disease at long-term follow-up [10].

Therefore, we aimed to study the usefulness of high BP in an ED as an opportunistic screening tool for undiagnosed hypertension.

## Patients and methods

### Study design and selection of patients

In this retrospective survey study, we aimed to include patients without diagnosed hypertension who had elevated BP during a visit at the ED. We used the local electronical medical system to identify all consecutive patients with acute internal medicine conditions that had attended the ED at Sahlgrenska University Hospital/Mölndal in Gothenburg, Sweden during a period of 6 months (from 2018-01-01 to 2018-03-31 and from 2018-07-01 to 2018-09-30). We included all patients with an obtained systolic BP ≥ 160 mmHg and/or diastolic BP ≥ 100 mmHg measured at the ED. We excluded patients with a previous diagnose of hypertension (identified by diagnose codes according to the International Classification of Disease 10th revision of I10-I15) registered in the electronical medical record. We also excluded patients on any BP-lowering medication (regardless reason), patients without Swedish social security number or deceased patients. Patients with recurrent BP measurements of <160/100 mmHg during index-hospitalization or at a recurrent visit to the ED within the same months were also excluded. All identified patients were contacted 2 years after their ED visit with a questionnaire regarding hypertension diagnosis. The study was approved by the Swedish Ethical Review Authority (#2020-00652 and 2021-01983) and informed consent was obtained from all participants.

### Setting

The ED at Sahlgrenska University Hospital/Mölndal is a tertiary ED open 24 h a day, every day of the year and also act as an academic teaching site. The ED assesses approximately 48,500 patients annually and receives patients above 15 years of age with internal medicine emergencies or orthopaedic emergencies.

### Methods of measurements

BP were measured routinely in the ED by a trained nurse or medical assistant using an automatic oscillometric device (Welch Allyn Connex Spot Monitor^91^71WX or Philips IntelliVue MX450). These devices are routinely calibrated by ED personnel according to the manufacturer’s specifications. BP values were mainly checked in a supine position or more rarely in a seated position. Values were registered either in the ED observational sheet or directly in the patients’ electronical medical record, by the nurse or the training assistant. Data on BP-values was extracted manually from either the observational sheet or the electronic medical records by three of the co-authors (OÖ, KA, SB). In case of multiple registered values, the lowest value was extracted. All methods were performed in accordance with relevant guidelines and regulations.

### Survey design

All identified patients were contacted two years after attending the ED, by mail with a letter of consent and a questionnaire with the following question:

1. Have you measured your BP after your visit to the ED (yes/no/free comment)?
2. Have you been diagnosed with hypertension (yes/no/free comment)?
3. If yes – what kind of BP-medication has been prescribed (free comment)?

All patients were given the chance, in case of raised questions regarding hypertension after receiving the letter, to contact a hypertension-specialised nurse or get a referral to a general practitioner. Patients not responding after the first letter were contacted again with a new letter after 2 months and by telephone 1 months further along. Patients not responding after that were considered declining to participate.

### Data analysis

The majority of the statistics were descriptive. Categoric variables are described with percentages, whereas continuous variables are reported with means and standard deviations (SDs) or medians and interquartile ranges, as appropriate. All analyses were conducted using Microsoft® Excel® version 2308.

## Results

**Fig. 1:**
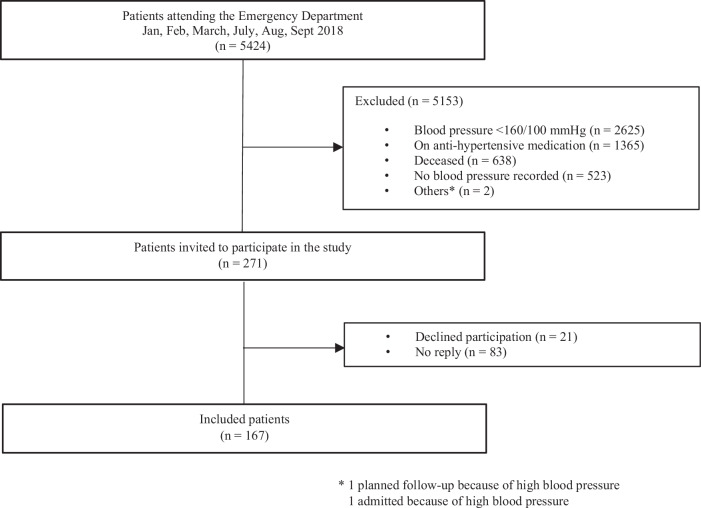
Flow chart.

5424 patients attended the ED during the 6-months-period. 2625 patients (48%) were excluded due to a BP < 160/100 mmHg, 1365 patients (25%) were excluded because of present BP-lowering medication, 523 patients (10%) were excluded because no BP was recorded, and 638 patients (12%) were deceased. A total of 271 patients were identified as suitable for the study and asked to participate. 167 individuals (62%) who agreed to participate, were included in the study, and responded to the questionnaire. For study flow chart see Fig. 1.

Mean age of the included patients (*n* = 167) was 63.1 years (SD ± 16.4) and 51% were women. Women were older, with a mean age of 66.5 years compared to 59.6 years of age for men. The mean systolic BP was 167.0 mmHg (SD ± 12.2), and the mean diastolic BP was 89.0 mmHg (SD ± 13.7). Men had a mean systolic BP of 167.3 mmHg (SD ± 12.7) and a mean diastolic BP of 91.1 mmHg (SD ± 16.0). Women had a mean systolic BP of 166.8 mmHg (SD ± 11.6) and a mean diastolic BP of 84.9 mmHg (SD ± 16.8). 14% (*n* = 23) had a systolic BP ≥ 180 and 6% (*n* = 10) had a diastolic BP ≥ 110 mmHg. Within this group none had signs of hypertensive crisis. For further baseline characteristics, see Table 1.

**Table 1:** Baseline characteristics of the study population.

*n*	167
Age, years ± SD	63.1 ± 16.4
Female	85 (50.9%)
SBP (mmHg) mean ± SD	167.0 ± 12.2
DBP (mmHg) mean ± SD	89.0 ± 13.7
Admitted to hospital	21 (12.6%)
Hypertensive treatment at the ED	2 (1.2%)
Comorbidity	
Coronary artery disease	5 (3.0%)
Heart failure	2 (1.2%)
Atrial fibrillation	11 (6.6%)
Cerebrovascular disease	6 (3.6%)
Peripheral artery disease	0 (0%)
Diabetes mellitus type 2	9 (5.4%)
Asthma/Chronic obstructive pulmonary disease	6 (3.6%)
History of cancer	14 (8.4%)
Rheumatic disease	3 (1.8%)

Data presented as numbers (n) and %, or mean values ± Standard deviation (SD); as appropriate.

*SBP* systolic blood pressure, *DBP* diastolic blood pressure, *ED* emergency department.

**Fig. 2:**
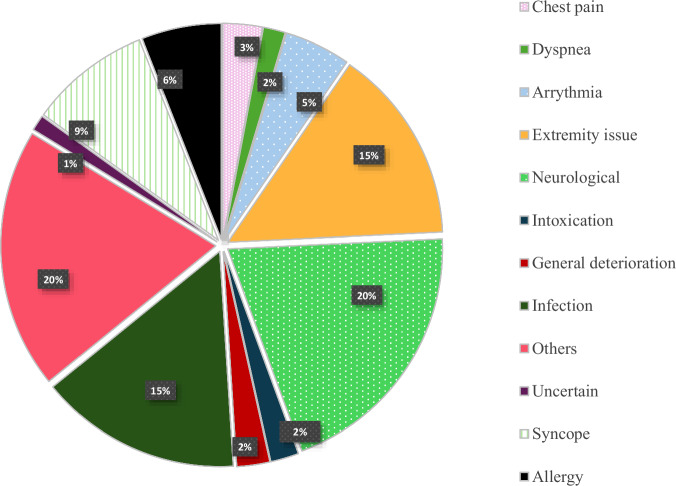
Reason for Emergency Department visit (*n* = 167).

The most common reason for emergency visit was neurological (20.1%), followed by miscellaneous (19.6%) and infections (15.2%), see Fig. 2. No patient had hypertension as a primary reason for the ED visit, however in 2 cases (1.2%) the clinicians chose to give blood pressure lowering treatment at the ED as well.

**Fig. 3:**
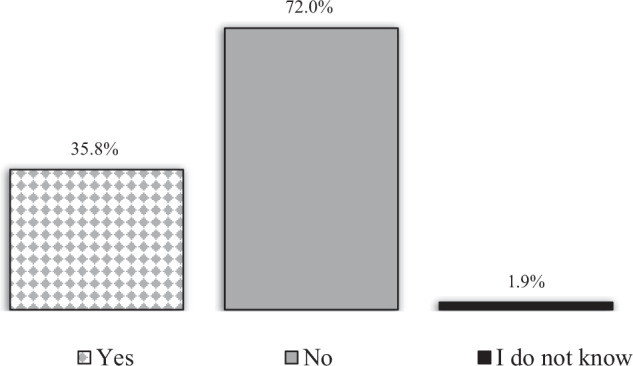
The answer frequency (*n* = 134) to question no. 2 “Have you been diagnosed with hypertension?”.

**Fig. 4:**
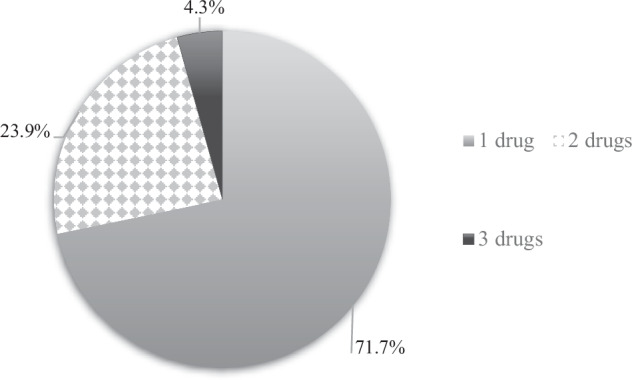
Numbers of blood pressure-lowering drugs in patients diagnosed with hypertension 2 years after attending the Emergency Department (question 3).

134 patients (80.2%) had measured their BP after the ED visit (question 1), and 48 (35.8%) of those had been diagnosed with hypertension (question 2), Fig. 3. 96% of patients diagnosed with hypertension were on BP-lowering medication and 24% were on 2 or more different BP-lowering agents (Fig. 4). The most common anti-hypertensive medication prescribed was RAAS-inhibitors (58.7%) followed by calcium-antagonists (43.5%), beta blockers (26.1%) and diuretics (4.3%).

## Discussion

This survey study followed 167 individuals without diagnosed hypertension, 2 years after a recorded systolic BP ≥ 160 mmHg and/or diastolic BP ≥ 100 mmHg at an ED visit. The main finding is that 80% of the population had measured their BP after the ED visit and one third of those had been diagnosed with hypertension. Thus, our results indicate that elevated BP during an ED visit, correlative to grade 2 hypertension levels, can reveal undiagnosed hypertension. This finding suggests the usefulness of following-up high BP after an ED visit in order to identify and treat individuals at risk.

We chose the screening cut off systolic BP value ≥ 160 mmHg and/or diastolic BP ≥ 100 mmHg as it is the defined cut off for grade 2 hypertension set by guidelines [11]. A higher cut off value, e.g. grade 3 hypertension (≥180/110 mmHg), would not be useful as those blood pressure levels, in clinical practice, generate a follow-up. A lower threshold, e.g. grade 1 hypertension (≥ 140/90 mmHg) could be a possible alternative, although it would most likely have resulted in a higher proportion of screening failure.

The current study confirms older, but smaller studies where 50-75% of patients with BP > 160/100 mmHg in the ED had BP levels indicative of hypertension using home BP monitoring [9, 12]. However, the small sample size in forementioned studies and using home BP monitoring might impact the differences with our results. Furthermore, in the current study the confirmed cases with hypertension had measured BP after the ED visit and were diagnosed with hypertension by a health-care professional. We believe that the robust form of self-reported diagnoses and reported prescription of medication is a strength in our investigation. Since we excluded patients with hypertension and/or anti-hypertensive medication our cohort showed a low percentage of other cardiovascular risk factors (only 3% had a registered history of coronary artery disease and 5% had diabetes mellitus) compared to other comparative studies [10, 13], as well as low admittance to in-hospital care (13%). Compelling, even in this “low-risk” population, subsequent hypertension was common, and our results expand earlier knowledge to this “low-risk”, unselected population.

Hypertension is highly prevalent, but up to half of the cases remain undiagnosed and not properly treated [1, 14]. Therefore, the current guidelines for the management of arterial hypertension from the European Society of Hypertension recommend opportunistic screening for hypertension in all adults [11]. However, due to limited resources in the health care system, screening young patients without risk factors does not gain wide implementation. Therefore, it is important to screen those patients when they contact the health care system, for instance at an ED visit. Elevated BP is common at the ED and depends often on the emergency context [8]. Acute pain leads to generalized increased sympathetic activity that increase the blood pressure. Transient high blood pressure per se is a pain-relieving action, possibly through increased endorphin release [15]. On the other hand, a lot of patients visiting the ED has undiagnosed hypertension that is masked in this stressful context.

Thus, using BP at the ED as an opportunistic screening for hypertension is debateable and confirming evidence of benefit are scarce. Many of the previous screening-studies do not include the ED as a potential source for screening [16]. Since measuring BP in the ED is routinely done, do not cause extra resources and is easily feasible, the ED might be a potential resource for screening. Our study shows a high rate of subsequent hypertension in patients with high BP in the ED, suggesting attention to an increased BP during an ED visit could potentially allow for earlier detection, better management, and prognosis for those patients.

Only 1% of the patients were treated with antihypertensive medication in the ED, despite a high mean BP. This might reflect the fact that physicians in the ED are not attentive to high BP or not aware of the importance of early detection of hypertension to minimize the risk for adverse cardiovascular events, including death [17].

Interestingly, we could see a high percentage of deceased patients, 12%, after two years. This is higher than reported earlier by McAllister, showing mortality rates of 3–5% within two years after an ED visit, in patients with high BP but no diagnose of hypertension [8]. However, our population were significantly older compared to that cohort, maybe explaining the surprisingly high mortality.

Some limitations in this study need to be addressed. First, the BP recorded at the ED were not standardized recording to recommendation by guidelines [11]. However, it was mainly recorded automatically, by trained nurses/assistant nurses and reflects clinical practice in an ED. Second, we lack information if the BP was taken in supine or seated position, potentially interacting with the results. Third, only 62% of the eligible patients agreed to participate in the study (167 out of the 271). This is, however, in line with positive response to previous contemporary studies [18]. Fourth, the retrospective follow-up design of the study might have affected the participation rate. In conclusion, this survey study shows that opportunistic screening at the ED of patients with BP ≥ 160/100 mmHg could within 2 years identify hypertension in one third of these individuals. This finding demonstrates the potential usefulness of BP measured at the ED as a screening instrument for hypertension.

## Summary

### What is known about the topic?

- Hypertension is the most preventable cause of morbidity and mortality, but many are underdiagnosed and lack treatment control.
- Hypertension in the Emergency Department is commonly observed, but mostly used for short term evaluation.
- The utility of blood pressure measured at the Emergency Department as a screening tool for undiagnosed hypertension is uncertain.

### What this study adds

- In patients without hypertension a blood pressure ≥160/100 mmHg at an ED visit can reveal undiagnosed hypertension in one third of the patients.
- Blood pressure measured at the emergency department might be used as a screening tool for hypertension.

## Data Availability

The data underlying this article will be shared on reasonable request to the corresponding author.
